# Cholesterol sensor SCAP contributes to sorafenib resistance by regulating autophagy in hepatocellular carcinoma

**DOI:** 10.1186/s13046-022-02306-4

**Published:** 2022-03-30

**Authors:** Danyang Li, Yingcheng Yao, Yuhan Rao, Xinyu Huang, Li Wei, Zhimei You, Guo Zheng, Xiaoli Hou, Yu Su, Zac Varghese, John F. Moorhead, Yaxi Chen, Xiong Z. Ruan

**Affiliations:** 1 Centre for Lipid Research & Key Laboratory of Molecular Biology for Infectious Diseases (Ministry of Education), Institute for Viral Hepatitis, Department of Infectious Diseases, the Second Affiliated Hospital, Chongqing Medical University, 400016 Chongqing, China; 2 Department of General Medicine, Affiliated Cancer Hospital of Chongqing University, Chongqing, 400016 China; 3 John Moorhead Research Laboratory, Centre for Nephrology, University College London Medical School, Royal Free Campus, University College London, London, NW3 2PF UK

**Keywords:** Sorafenib resistant, SCAP, Autophagy, Lycorine, Hepatocellular carcinoma (HCC)

## Abstract

**Background:**

Hepatocellular carcinoma (HCC) is one of the most malignant tumors and the fourth leading cause of cancer-related death worldwide. Sorafenib is currently acknowledged as a standard therapy for advanced HCC. However, acquired resistance substantially limits the clinical efficacy of sorafenib. Therefore, further investigations of the associated risk factors are highly warranted.

**Methods:**

We analysed a group of 78 HCC patients who received sorafenib treatment after liver resection surgery. The expression of SCAP and its correlation with sorafenib resistance in HCC clinical samples were determined by immunohistochemical analyses. Overexpression and knockdown approaches in vitro were used to characterize the functional roles of SCAP in regulating sorafenib resistance. The effects of SCAP inhibition in HCC cell lines were analysed in proliferation, apoptosis, and colony formation assays. Autophagic regulation by SCAP was assessed by immunoblotting, immunofluorescence and immunoprecipitation assays. The combinatorial effect of a SCAP inhibitor and sorafenib was tested using nude mice.

**Results:**

Hypercholesterolemia was associated with sorafenib resistance in HCC treatment. The degree of sorafenib resistance was correlated with the expression of the cholesterol sensor SCAP and consequent deposition of cholesterol. SCAP is overexpressed in HCC tissues and hepatocellular carcinoma cell lines with sorafenib resistance, while SCAP inhibition could improve sorafenib sensitivity in sorafenib-resistant HCC cells. Furthermore, we found that SCAP-mediated sorafenib resistance was related to decreased autophagy, which was connected to decreased AMPK activity. A clinically significant finding was that lycorine, a specific SCAP inhibitor, could reverse acquired resistance to sorafenib in vitro and in vivo.

**Conclusions:**

SCAP contributes to sorafenib resistance through AMPK-mediated autophagic regulation. The combination of sorafenib and SCAP targeted therapy provides a novel personalized treatment to enhance sensitivity in sorafenib-resistant HCC.

**Supplementary Information:**

The online version contains supplementary material available at 10.1186/s13046-022-02306-4.

## Background

Virus-associated hepatocellular carcinoma (HCC) has decreased year by year due to the widespread use of the HBV vaccine and therapeutic HCV treatment. However, non-alcoholic steatohepatitis (NASH) has gradually become an important driver of HCC. Potentially curative treatments for HCC, such as liver transplantation, tumour resection, or ablation, are limited to early-stage tumours. Sorafenib is a tyrosine kinase inhibitor that exhibits angiogenic and proliferative effects and is widely used in advanced HCC treatment [1]. Sorafenib was reported to prolong the median overall survival (OS) by 2.3 − 3 months in advanced HCC patients [2, 3]. Unfortunately, many HCC patients have a poor response to sorafenib or develop resistance to sorafenib treatment within 6 months [4, 5]. Thus, it is important to invent new drugs and/or develop a novel treatment strategy that increases the efficacy of sorafenib.

Hypercholesterolaemia is a major risk factor for cardiovascular diseases [6]. A growing number of studies have revealed that hypercholesterolaemia is related to cancer progression and prognosis [7, 8]. In hepatocellular carcinoma (HCC) subjects, hypercholesterolemia was documented as paraneoplastic syndromes leading to poor survival rates [9]. Dietary cholesterol caused spontaneous NAFLD–HCC formation by cholesterol-induced gut microbiota changes and metabolomic alterations [10]. A recent retrospective study showed that statin use was associated with decreased liver cancer mortality when adjusting for cholesterol levels [11]. Moreover, cholesterol can affect the functional outcome of anticancer drugs in various cancer cell types [12, 13]. However, the relationship between hypercholesterolaemia and sorafenib resistance in HCC remains an open question.

The liver is the primary site for the regulation of serum cholesterol via modulating their biosynthesis and metabolism as well as packaging, reuptake, and export of lipoproteins [14]. The derangements in hepatic cholesterol metabolism can lead to metabolic disorders, such as hypercholesterolemia [15]. The sterol-regulatory element binding protein (SREBP) cleavage-activating protein (SCAP) is a cholesterol sensor and chaperone of SREBPs that maintains a constant level of intracellular cholesterol [16, 17]. Apart from the mutations in LDLR and apolipoprotein B (ApoB), the gain of function mutation in SCAP or SREBP2 gene is implicated in autosomal dominant familial hypercholesterolemia [18]. Recent studies have shown that SCAP may be associated with the sensitivity to antitumour treatment. Deletion of SCAP in intratumoral regulatory T cells (Treg cells) inhibited tumour growth. It enhanced the sensitivity to immunotherapy through a process that depends on SREBP activity and signals via mevalonate metabolism to protein geranylgeranylation [19]. Based on these data, in the present study, we sought to determine the role of SCAP in sorafenib resistance.

Recent studies have revealed the roles of epigenetics (OCT1 methylation [20]), transport processes (ABCC2 variants [21]), cell death regulation (autophagy [22]), and the microenvironment of cancer (hypoxia [23]) in primary and acquired resistance to sorafenib in HCC [24]. Autophagy is the classic mechanism of resistance to sorafenib [25]. During HCC therapy, sorafenib induces autophagy, which promotes the ability of sorafenib to kill HCC cells [26]. Enhancing autophagy could increase the antiproliferative effects of sorafenib by trehalose [27]. Conversely, silencing key autophagic pathway genes diminished the antiproliferative effects of sorafenib. In addition, there is a complex interaction between autophagy and lipid metabolism. Autophagy was also shown to contribute to cholesterol ester hydrolysis through a mechanism of cholesterol metabolism termed "lipophagy" [28]. Lipophagy was originally described in hepatocytes and has constantly been correlated with the accumulation of lipids and lipid droplets in vitro and in vivo [29]. The key energy sensor AMPK has been shown to be an important modulator of autophagy [30]. Interestingly, our previous work demonstrated that excessive lipid deposits inhibit AMPK activity and that SCAP negatively regulates AMPK activity, while SCAP knockdown could reduce lipid accumulation, consequently increasing autophagy by activating AMPK [31]. Therefore, we hypothesize that SCAP modulates autophagy by regulating the activity of AMPK, which may be the underlying mechanism and a novel therapeutic target for the reversal of sorafenib resistance.

In this study, we demonstrated that SCAP is overexpressed and cholesterol is deposited in sorafenib-resistant HCC tissues/cells, and silencing SCAP substantially increases the sensitivity of HCC cells to sorafenib treatment. Mechanistic studies revealed that inhibition of SCAP reverses sorafenib resistance by promoting autophagy through activation of the phosphorylation of AMPK. Moreover, animal xenograft models showed that the combination of the specific SCAP inhibitor lycorine and sorafenib was sufficient to inhibit tumour growth, suggesting that targeting SCAP may be a new therapeutic strategy for sorafenib-resistant HCC.

## Materials and methods

### Materials

Sorafenib, lycorine, Desmosterol, Compound C and chloroquine were purchased from MedChemExpress (Monmouth Junction, NJ, USA).

### Cell culture

Human HCC cell lines PLC/PRF/5, HepG2 were obtained from the American Type Culture Collection (ATCC, VA, USA); MHCC-97H, SK-Hep1, and Huh7 from the Cell Bank of the Chinese Academy of Sciences (Shanghai, China). Sorafenib-resistant clones were established by subjecting PLC/PRF/5 and MHCC-97H cells to continuous administration of gradually increasing Sorafenib concentrations and trained up to 8 μM Sorafenib. Cells were maintained in high glucose DMEM supplemented with 10% fetal bovine serum (FBS), 100 mg/mL of streptomycin, and 100 unit/mL of penicillin at 37 °C in 5% CO2. LDL was isolated from the plasma of healthy human volunteers by sequential ultracentrifugation. Informed consent (ethical approval: Sheffield REC 10/H1308/25 according to the principles outlined in the Declaration of Helsinki) was obtained from volunteers regarding the use of their plasma samples for research.

### Patients

Datum of cases (78 cases) was obtained from randomly selected Sorafenib-treated HCC patients who under-treated at the Second Affiliated Hospital of Chongqing Medical University and Chongqing University Cancer Hospital between 2015 and 2021, with the approval of the Institutional Review Board of Chongqing Medical University and Chongqing University Cancer Hospital.

### Patient samples

HCC tissues were obtained from 24 patients who under Sorafenib treatment at the Second Affiliated Hospital of Chongqing Medical University and Chongqing University Cancer Hospital between 2015 and 2021, with the approval of the Institutional Review Board of Chongqing Medical University. Patients provided informed consent. All specimens were frozen immediately after surgery and stored in liquid nitrogen until use.

### Cell proliferation assay

Cells were seeded in 96-well plates at a density of 5000 cells/well. After 24 h, the cells were incubated in a serum-free medium for 12 h. Then, the cells were subjected to a concentration gradient of Sorafenib for 48 h. All experiments were carried out in a serum-free DMEM medium. The OD values were measured at 450 nm after incubation with CCK-8 reagent for 1 to 2 h at 37 °C.

### Oil red o staining

Intracellular lipids were stained by means of Oil Red-O (Solarbio Life Science, Beijing, China). Cells were washed with phosphate-buffered saline (PBS) and fixed with 4% paraformaldehyde in PBS for 10 min. Fixed cells were incubated with Oil Red-O solution for 20 min at room temperature and then with 4',6-diamidino-2-phenylindole (DAPI) (Solarbio Life Science, Beijing, China) for 5 min. Frozen slices from euthanized mice were permeabilized with 4% paraformaldehyde for 20 min. After rinsing with PBS for 5 min approximately 3 times, the slices were stained with Oil Red O for 15 min at 37° C. Next, the slices were washed with ddH2O and counterstained with 4',6-diamidino-2-phenylindole (DAPI) for 8 min. Finally, all the slides were examined under a light microscope.

### Lipid analysis

Serum total cholesterol (TC), Serum triglycerides (TG) and total intracellular cholesterol (TC) were tested on enzymatic colorimetric methods using commercial kits purchased from the Nanjing Jiancheng Bioengineering Institute (Nanjing, China).

### Western blot analysis

Protein lysates of cells or tumor tissues were extracted by RIPA lysis buffer (Beyotime Biotechnology, Jiangsu, China). Equal volumes of protein samples were separated by 7.5% or 12.5% SDS/PAGE and electro-transferred to PVDF membranes (Millipore, Billerica, MA, USA). The immunoblots were probed with the indicated antibodies. Finally, the detection was performed using an ECL chemical luminescent detection kit (Bio-Rad), and the bands were further analyzed using ImageJ software. The expression of the target protein was normalized to β-actin expression.

### Real-time quantitative PCR (qRT-PCR)

Total RNA was extracted using TRIzol reagent (Takara) and reverse transcribed into cDNA. Next, the cDNA products were subjected to 2-step PCR amplification. The relative expression of the genes was analyzed using the 2-∆∆ Ct method, and β-actin was used as the internal reference gene.

### Gene silencing

Knockdown of SCAP in cells was achieved by using a reverse siRNA transfection procedure performed in six-well plates. Therefore, for each well to be transfected, 5 μl Lipofectamine RNAiMAX (Thermo Fisher Scientific, Eugene, OR) was mixed with 500 μl Opti-MEM (Thermo Fisher Scientific) and combined with 25 pmol siRNA (GenePharma, Shanghai, China). The transfection mixture was incubated at room temperature for 20 min. Cells were harvested in a complete growth medium without antibiotics and diluted so that 2 ml contained the appropriate number of cells to give 30% to 50% confluence 24 h after plating. Cell suspensions were mixed with the transfection mixture and incubated.

### Colony formation assay

Cells were plated in 6-well plates at a density of 4000 cells/well with a medium containing 10% FBS. Then, the cells were treated with Sorafenib (6 μM) for 48 h and plated for 2 weeks. Colonies were fixed and stained with a 0.1% crystal violet solution and counted grossly.

### Wound healing

Cells were seeded in 6-well plates and treated with Sorafenib (6 μM) for 48 h, and the monolayer was scratched with a pipette tip. After that, the cells were incubated in a serum-free medium for 0 to 72 h. Then, the wound areas were quantified using Image J software.

### Transwell assays

For the transwell migration assays, cells in the upper chamber were treated with Sorafenib (6 μM) for 48 h in advance, while DEME containing 10% FBS was added to the lower chambers. For the transwell invasion assays, the upper membrane was coated with 50 μl Matrigel (BD Biosciences) in advance. After incubation, the cells were fixed and stained with trypan blue.

### Flow cytometry analysis

Following treatment, cells were collected and resuspended in 1 ml of ice-cold PBS at a density of 1 × 10^6^ cells/mL or fixed with 75% alcohol, and the samples were immediately detected by flow cytometry (BD Biosciences, US) and the data were analyzed using FlowJo software version 10.

### Transmission electron microscopy (TEM)

Fresh tissue and cells were placed in 4% glutaraldehyde overnight at 4 °C. Ultrathin sections were cut and then stained. Images were acquired on a transmission electron microscope. For the quantification of autophagy, autophagic vacuoles (defined as autophagosomes, double-membraned structures surrounding cytoplasmic material, and autolysosomes, lysosomes containing cytoplasmic material) were counted.

### Autophagic flux analysis

Cells were first transduced with siSCAP or siVector in a confocal dish. 24 h after the first transduction, the cells were then transduced with monomeric red fluorescent protein (mRFP)-GFP-LC3 adenoviral vectors (HanBio Technology, Shanghai, China). The principle of the assay is based on the different pH stability of red and green fluorescent proteins. The enhanced GFP signal could be quenched under the acidic condition (pH < 5) inside the lysosome, whereas the mRFP signal did not change significantly in acidic conditions. In red- and green-merged images, autophagosomes are shown as yellow puncta, while autolysosomes are shown as red puncta. An enhancement of both yellow and red puncta in cells indicate that autophagic flux is increased, while autophagic flux is blocked when only yellow puncta are increased without alteration of red puncta, or when both yellow and red puncta are decreased in cells. Cells were incubated in 1 ml growth medium with the adenoviruses for 2 h at 37 °C, and the growth medium was replaced with fresh medium. Cells were treated with Sorafenib (6 μm) at the same time. Experiments were performed 48 h after the second transduction. LC3 puncta were examined with a Leica confocal microscope.

### Animal model and treatment

Animal care and experimental procedures were performed with approval from the Animal Care Committee of Chongqing Medical University. All animal studies were conducted in accordance with institutional guidelines for the care and use of experimental animals. Animal experiments were conducted using 4–6-week-old BALB/c nude mice (male, 20–25 g). Mice were purchased from Gempharmatech Co., Ltd. (Nanjing, China). For the xenograft implantation model, a total of 20 nude mice were randomly divided into 4 groups, including control (con), lycorine (Ly), Sorafenib (Sora) and lycorine + Sorafenib (Ly + Sora). 2 × 10^6^ cells were subcutaneously injected into the flanks of the mice. Tumor growth was measured every 2 days, and the volumes of the xenograft tumors were calculated using the following standard formula: length × width × width × 0.5. Treatment was initiated on the fifth day, tumor-bearing mice were intragastric administration with lycorine (10 mg/kg/day, 2.5 mg/mL in 5% dimethyl sulfoxide (DMSO)), Sorafenib (30 mg/kg/day, 1.2 mg/mL in 5% DMSO), both, or vehicle (5% DMSO dissolved in saline) (*n* = 5 per group) for 30 days. After 30 days, the mice were sacrificed, and tumor tissues were harvested for histological analysis.

### Immunofluorescent staining

Frozen slices or cells were fixed with 4% paraformaldehyde for 15 min and incubated with 0.3% Triton X100 for 15 min. After blocking with 3% bovine serum albumin, the slices were incubated with the following primary antibodies: anti-p-AMPK (1:100, CST), anti-P62(1:200 proteintech). After overnight incubation, the slices or cells were then incubated with fluorescence-conjugated secondary antibodies for 1 h. Finally, the slices or cells were incubated with Hoechst for 5 min, and then images were captured under a Zeiss fluorescence microscope and analyzed using Image Pro Plus software.

### Immunohistochemistry (IHC) staining

Tumor tissue samples were fixed in 4% paraformaldehyde and embedded in paraffin according to standard procedures. Sections were incubated with the indicated primary antibodies overnight at 4 °C. Subsequently, the slides were incubated with a secondary anti-rabbit or anti-mouse IgG (ZSGB-BIO, Beijing, China) and visualized using 3,3′-diaminobenzidine (ZSGB-BIO). Stained slides were scanned with a Pannoramic Scan 250 Flash or MIDI system and images were acquired using Pannoramic Viewer 1.15.2 (3DHistech, Budapest, Hungary). Images were analyzed using Image Pro Plus software.

### Study approval

For patient samples, the study protocol was approved by the Medical Ethics Committee of Chongqing Medical University. Patients were provided informed consent. All experimental procedures performed on animals were approved by Institutional Animal Care and Use Committee at the Chongqing Medical University (license number: 2017011). All mice were maintained under specific pathogen-free conditions in the laboratory animal center of Chongqing Medical University. Animal care and use protocols adhere to National Regulations for the Administration of Laboratory Animal to ensure minimal suffering.

### Statistical analysis

All data were presented as means ± SD. Sample sizes for relevant experiments were determined by power analyses conducted during experiment planning. Appropriate statistical analyses were performed using GraphPad Prism 5.0 software (GraphPad Software Inc, La Jolla, CA, USA). Statistical significance was determined using one-way ANOVA for multiple comparisons. Student’s t-test was used to compare two groups. Pearson correlation was used to analyze the relationship between SCAP protein expression and sensibility of Sorafenib. The chi-square test and Student’s t-test was applied to determine the association between clinicopathological parameters and tumor development. Probability values (*p*) < 0.05 were considered statistically significant.

## Results

### Clinical study

**Fig. 1:**
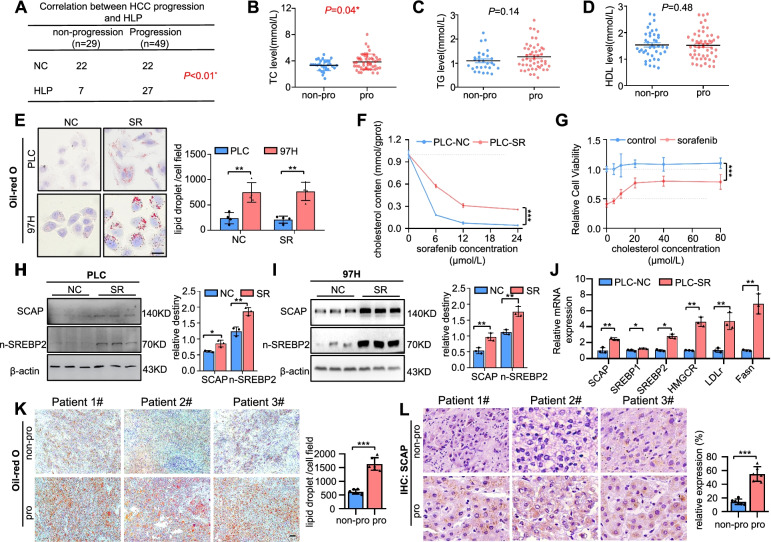
The levels of SCAP are elevated in sorafenib-resistant HCC cells. Correlation between cancer progression and HLP (**A)**, TC level (**B)**, TG level (**C)**, and HDL level (**D)** (*n* = 78). **E** Representative images of parental and SR cells stained with Oil Red O (*n* = 4). Bar = 50 μm. **F** Cholesterol content in parental cells and PLC-SR cells treated with sorafenib with a certain concentration gradient (*n* = 3). **G** The viability of sorafenib-treated cells with the addition of cholesterol in a dose-dependent manner. Immunoblot analysis of SCAP and n-SREBP2 protein expression in parental and PLC-SR cells (**H)** and 97H-SR cells (**I)** (*n* = 3). **J** mRNA expression of SCAP and downstream genes (SREBP1, SREBP2, HMGCR, LDLr, Fasn) as measured by qRT-PCR (*n* = 3). **K** Representative images of HCC tissues stained with Oil Red O (*n* = 6). Bar = 50 μm. **L** IHC staining of SCAP in HCC tissues (*n* = 6). Bar = 50 μm. Data are the mean ± SD. *P < 0.05, **P < 0.01, ***P < 0.001. P values were determined by chi-square tests in (**A**); Student’s t test in (**B**), (**C**), (**D**), (**E**), (**H**), (**I**), and (**J**); and repeated-measures ANOVA in (**F**) and (**G**)

We analysed a group of 78 HCC patients who received sorafenib treatment after liver resection surgery from 2015 to 2021 (Table 1). The patients were dichotomized into two groups based on whether the disease progressed after sorafenib treatment. There was no significant correlation between sorafenib resistance and other clinicopathological features, such as age (*P* = 0.83), body mass index (BMI) (*P* = 0.74), and drinking status (*p* = 0.2). In contrast, the strongest positive correlation was observed with hyperlipidaemia (24.1% vs. 55.1%, *P* < 0.01) (Fig. 1A). We further meticulously evaluated total cholesterol (TC), triglyceride (TG), and high-density lipoprotein (HDL) levels in the progressed patients (*n* = 49) compared with the patients with nonprogression (*n* = 29) (Fig. 1B-D. Table 1). Plasma TC, rather than TGs and HDL, was higher in the progression group, suggesting that elevated plasma TC may be a key risk factor in sorafenib-resistant HCC.

**Table 1:** clinical characteristics and blood lipid level in non-progressive and progressive HCC patients with sorafenib treatment

	Non-progression (*n* = 29)	Progression (*n* = 49)	P
Age(years)	53.17 ± 11.81	53.71 ± 9.78	0.83
Male rate(%)	93.10%	73.47%	0.03*
Drink rate(%)	31.03%	18.37%	0.20
BMI(Kg/m^2^)	22.64 ± 2.91	22.40 ± 3.13	0.74
TG(mmol/L)	1.10 ± 0.42	1.27 ± 0.53	0.14
TC(mmol/L)	4.17 ± 1.04	4.81 ± 1.48	0.04*
HDL(mmol/L)	1.43 ± 0.41	1.53 ± 0.53	0.48
LDL(mmol/L)	2.37 ± 0.72	2.70 ± 1.18	0.18
AFP(ng/ml)	295.24 ± 408.51	334.80 ± 435.48	0.70
ALT(U/L)	49.82 ± 47.00	55.02 ± 50.37	0.66
AST(U/L)	69.52 ± 69.83	74.34 ± 76.09	0.78
ALB(U/L)	39.46 ± 8.00	39.29 ± 7.81	0.93

*BMI* BodyMassIndex, *TC* Total cholesterol, *TG* Total triglyceride, *HDL* High-density lipoprotein cholesterol, *LDL* Low-density lipoprotein cholesterol, *AFP* Alpha-fetal protein, *ALT* Glutamic-pyruvic transaminase, *AST* Glutamic-oxalacetic transaminase, *ALB* Albumin

Non-progression group: there is no medical imaging progress after a period treatment of sorafenib progression group: there is medical imaging progress after a period treatment of sorafenib

### The levels of SCAP are elevated in sorafenib-resistant HCC cells

To investigate the molecular mechanism of sorafenib resistance linked to hypercholesterolaemia in HCC, we generated two sorafenib-resistant (SR) HCC cell lines in vitro. First, we examined the sensitivity of five types of HCC cell lines, HepG2, 97H, SK, Huh7, and PLC. The results showed that the PLC and HepG2 cell lines were the most and the least sensitive, respectively (Fig. S1A). We selected the two relatively more sensitive cell lines, 97H and PLC, for further study. We gradually increased the concentration of sorafenib in the medium over repeated passages to achieve resistance (Fig. S1B). We confirmed the acquired resistance of these two resistant cell lines by comparing them to their parental cell lines and named them PLC-SR and 97H-SR. The half-maximal inhibitory concentrations (IC50) of PLC-SR and 97H-SR cells to sorafenib were over 2 times higher than those of the parental cells at 14.296 and 11.754 μM, respectively (Fig. S1C-D).

Considering the association of hypercholesterolaemia with sorafenib-resistant HCC, we observed whether lipids accumulated and quantified the total cholesterol in the PLC-SR and 97H-SR cells (Fig. 1E-F). Increased intracellular cholesterol levels were observed in the SR cells, and the increased cholesterol might rescue PLC cells from death by sorafenib, suggesting that excessive intracellular cholesterol accumulation in HCC cells may be one of the key reasons for sorafenib resistance. Since the SCAP/SREBP2 pathway is closely linked to cholesterol metabolism and hypercholesterolaemia, we further detected the signalling pathway. The data showed that SCAP/SREBP2 mRNA and protein levels were elevated in PLC-SR and 97H-SR cells. Then, the mRNA and protein levels of SCAP were detected in the abovementioned five types of HCC cell lines. There was a close and significant correlation between SCAP expression and resistance to sorafenib (R^2^ = 0.6922, *P* = 0.0001) (Fig. S1E-H). The association between decreased sorafenib sensitivity and increased SCAP expression and lipid deposition was corroborated by the results of ORO and IHC assays of human HCC tissues (Fig. 1K-L). In summary, these data indicate that SCAP plays positive roles in mediating sorafenib resistance in HCC cells.

### Disorders of the SCAP signalling pathway trigger sorafenib resistance in HCC cell lines

We further tried to determine whether the overexpression of SCAP would induce sorafenib resistance in HCC cells. SCAP signalling activity is normally under tight metabolic control through a feedback system dependent on intracellular cholesterol concentration. This system maintains stable cholesterol levels to control both the rate of cholesterol uptake via LDL and the rate of cholesterol synthesis in hepatocytes and other cells [32]. A D443N point mutation was generated in the sterol-sensitive domain of SCAP, facilitating SCAP-SREBP complex translocation and sterol resistance [33]. Interestingly, we found that SCAP^D443N^ overexpression in PLC observably increased sorafenib resistance (Fig. S2A-C).

**Fig. 2:**
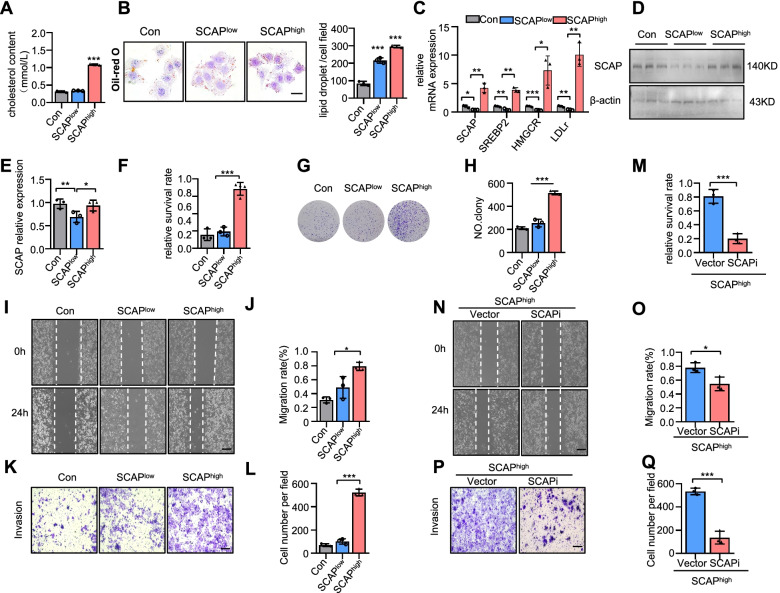
Disorders of the SCAP signalling pathway trigger sorafenib resistance in HCC cell lines. Loading LDL (100 ng/ml) and IL-1β (20 ng/ml) for 24 h simulated lipid disorders in HCC patients. The untreated group was defined as the control group. The LDL group was defined as SCAP^low^ group. The LDL and IL6 group was defined as SCAP^high^ group. **A** Cholesterol contents in the SCAP^low^ and SCAP^high^ cells (*n* = 3). **B** Representative images of each group stained with Oil Red O (*n* = 3). Bar = 50 μm. **C** mRNA expression of SCAP and downstream genes (SREBP2, HMGCR, LDLr) as measured by qRT-PCR (*n* = 3) in the SCAP^low^ and SCAP^high^ cells (*n* = 3). **D** Immunoblot analysis of SCAP protein expression (*n* = 3). **E** The histogram represents the relative expression of SCAP. **F** The viability of sorafenib-treated cells with the above treatments. **G**, **H** Clone assays of cells with the above treatment (*n* = 3). Scratch-wound cell migration assays (**I**) and invasion assays (**K**) of cells with the above treatment (*n* = 3). Bar = 100 μm. The histogram represents the distance of cell migration (**J**) and invasion (**L**) in each group. Knockdown of SCAP in the SCAP^high^ groups using a reverse siRNA transfection procedure. **M** The viability of sorafenib-treated vector and SCAP-knockdown cells (*n* = 3). Scratch-wound cell migration (**N**) and invasion (**P**) assays for the vector and SCAP-knockdown cells (*n* = 3) Bar = 100 μm. The histogram represents the distance of cell migration (**O**) and invasion (**Q**) in each group. Data are the mean ± SD. *P < 0.05, **P < 0.01, ***P < 0.001. P values were determined by one-way ANOVA in (**A**), (**D**), (**F**), (**G**), (**I**), (**K**), (**L**), (**N**), and (**P**) and repeated-measures ANOVA in (**B**)

Our previous study demonstrated that LDL loading decreases SCAP expression, but LDL loading plus IL-1β increases SCAP expression and disrupts normal SCAP trafficking between the ER and Golgi, which induces SREBP2, HMGCR, and LDLr expression in HepG2 cells [34]. The same observations were also found in the PLC cell line (Fig. 2A-E). Therefore, we named the LDL group SCAP^low^ and the IL-1β + LDL group SCAP^high^. Consistent with the target gene expression patterns, PLC cells presented a higher survival rate and SCAP^high^ PLC cells formed enlarged colonies (Fig. 2F-H). Wound healing and Transwell assays showed that SCAP^high^ PLC cells displayed partially restored migration and invasion under sorafenib treatment for 24 h (Fig. 2I-L). In contrast, desmosterol (DES), an ER-Golgi transport inhibitor, reduced SCAP Golgi accumulation and significantly improved sorafenib sensitivity (Fig. S3A-B). These data suggested that an imbalance in cholesterol homeostasis reduces the sensitivity of HCC cells to sorafenib. To determine whether SCAP plays a role in this process, we induced knockdown of SCAP in SCAP^high^ PLC cells via the siRNA system and observed increased the sensitivity to sorafenib in HCC cells and inhibited cell proliferation, migration and invasion (Fig. 2M-Q).

### SCAP is vital for sustaining sorafenib resistance

**Fig. 3:**
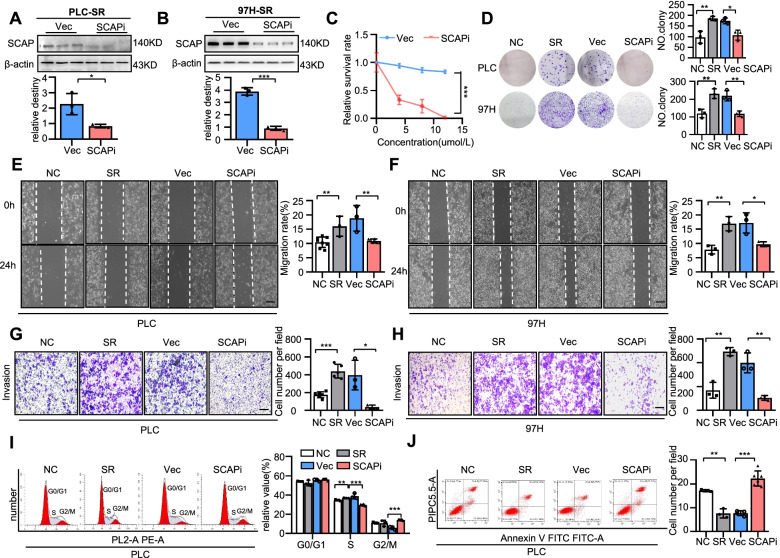
SCAP is vital for sustaining sorafenib resistance. Knockdown of SCAP in sorafenib-resistant PLC/PRF/5 and MHCC-97H cells using a reverse siRNA transfection procedure. Immunoblot analysis of SCAP protein expression in PLC (**A**) and 97H (**B**) cells (*n* = 3). **C** The viability of sorafenib-treated PLC vector cells and SCAP knockdown cells (*n* = 3). Parental cells (NC), sorafenib-resistant cells (SR), SR vector cells (Vec) and SR SCAP knockdown cells (SCAPi) were treated with sorafenib (6 μm/L) for 24 h. **D** Clone assays of 4 groups in PLC and 97H cells (*n* = 3). Scratch-wound cell migration assays for each group of PLC (**E**) and 97H cells (**F**) (*n* = 3). Invasion assays for each group of PLC (**G**) and 97H cells (**H**) (*n* = 3). Bar = 100 μm. Flow cytometry analysis of the cell cycle (**I**) and apoptosis (**J**) of PLC cells after exposure to sorafenib (6 μm/L) for 24 h (*n* = 3). Data are the mean ± SD. *P < 0.05, **P < 0.01, ***P < 0.001. P values were determined by Student’s t test in (**A**) and (**B**); repeated-measures ANOVA in (**C**) and (**I**); and one-way ANOVA test in (**D**), (**E**), (**F**), (**G**), (**H**), and (**J**)

To further confirm the function of SCAP in sorafenib resistance, we used SCAP knockdown by siRNA in HCC-SR cells. The knockdown efficiency of siRNA in two sorafenib-resistant cell lines was proven (Fig. 3A-B). We next examined the effect of silencing SCAP on sorafenib resistance. As reflected by cell viability assays, depletion of SCAP by RNAi markedly reversed sorafenib resistance in two sorafenib-resistant HCC cell lines, as shown in Fig. 3C. Similar results were found in colony formation assays (Fig. 3D). In addition, we assessed the effect of SCAP inhibition on the migration and invasion of sorafenib-resistant HCC cells. Similarly, wound healing and Transwell assays showed that suppression of SCAP expression inhibited the migration and invasion of two sorafenib-resistant cell lines (Fig. 3E-H). Next, cell cycle analysis showed that the percentage of cells in the S phase was decreased and that of cells in the G2 phase was increased by SCAP siRNA (Fig. 3I). The potent efficacy of SCAP suppression was also demonstrated by a cell apoptosis assay through flow cytometry (Fig. 3J). These data indicate the important role of SCAP in sorafenib resistance in HCC cells.

### SCAP regulates autophagy in HCC by influencing AMPK signalling

**Fig. 4:**
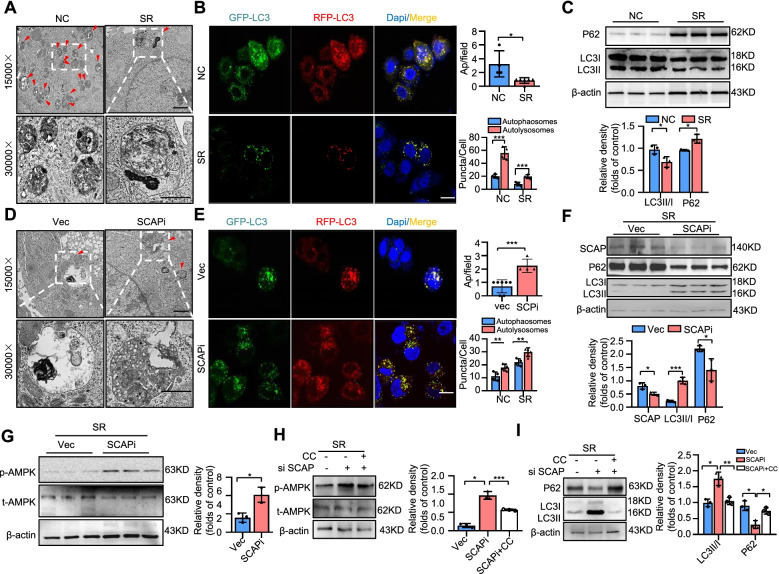
SCAP regulates autophagy in HCC by influencing AMPK signalling. Parental PLC cells (NC) and PLC-SR cells (SR) were treated with sorafenib (6 μm/L) for 24 h. **A** Ultrastructural analysis of parental cells and PLC-SR cells. The red arrowhead represents autophagic vacuoles (defined to include autophagosomes and autolysosomes) (*n* = 3). Bar = 50 μm. **B** Fluorescence and quantification of LC3-positive autolysosomes or autophagosomes in each group (*n* = 3). Bar = 100 μm. **C** Immunoblot analysis of LC3 and P62 protein expression in each group (*n* = 3). Vector cells (Vec) and SCAP knockdown cells (SCAPi) were treated with sorafenib (6 μm/L) for 24 h. **D** Ultrastructural analysis of SR vector cells and SR SCAP knockdown cells. The red arrowhead represents autophagic vacuoles (defined to include autophagosomes and autolysosomes) (*n* = 3). **E** Fluorescence and quantification of LC3-positive autolysosomes or autophagosomes in each group (*n* = 3). **F** Immunoblot analysis of SCAP, LC3 and P62 protein expression in each group (*n* = 3). **G** Immunoblot analysis of p-AMPK and t-AMPK protein expression in each group (*n* = 3). SR SCAP knockdown cells were treated with the phosphorylation inhibitor Compound C (SCAPi + CC) (4 μm/L) for 12 h, and Vec cells, SCAPi cells and SCAPi + CC cells were treated with sorafenib (6 μm/L) for 24 h. Immunoblot analysis of p-AMPK, t-AMPK (H), LC3 and P62 (I) protein expression in each group (*n* = 3). Data are the mean ± SD. *P < 0.05, **P < 0.01, ***P < 0.001. P values were determined by Student’s t test in (**A**), (**B**), (**C**), (**D**), (**E**), (**F**), and (**G**) and one-way ANOVA in (**H**) and (**I**)

Previous reports have shown that chemoresistance in human cancers is associated with autophagy [20]. Our results supported this conclusion that sorafenib killed cells accompanied by autophagic activation (Fig. S4A-B). We first detected the levels of autophagy in PLC-SR cells and parental cells. Morphological changes were observed by transmission electron microscopy (TEM), and the results showed a lower number of autophagosomes in the PLC-SR cells than in the parental cells (Fig. 4A). RFP-GFP-tagged LC3 was used to monitor autophagosomes via fluorescence assays. Based on the theory that the GFP signal is sensitive to acidic and/or proteolytic conditions, RFP is more stable. We found that PLC-SR cells had lower levels of autophagy than the parental cells (Fig. 4B). A hallmark of autophagy is the transformation of the soluble form of LC3 (LC3-I) to a lipidated and autophagosome-associated form (LC3-II). P62 is also an autophagic receptor that accumulates in cells when autophagic flux is inhibited. To further confirm this phenomenon, we measured the levels of P62 and lipidation of LC3. In PLC-SR cells, the levels of LC3-II were decreased and the levels of P62 were enhanced (Fig. 4C, S5A). These data were consistent with previous reports.

To determine whether SCAP regulation played a role in autophagy to mediate sorafenib resistance in HCC, we performed a series of experiments in PLC-SR cells with or without SCAP depletion by siRNA and found the same results. Our results showed that knockdown of SCAP significantly increased the number of autophagosomes and LC3-II accumulation and decreased the levels of P62 in PLC-SR cells (Fig. 4D-F, S5B), suggesting ongoing autophagy. We next used chloroquine (CQ), a compound that inhibits autophagic flux by decreasing autophagosome-lysosome fusion, caused a remarkable increasing in the survival rate (Fig. S5C). We and others have previously shown that SCAP expression levels are linked to AMPK activity, which can directly modulate autophagy [31], so we next examined the expression levels of AMPK and phosphorylated AMPK (p-AMPK) in vitro. As we anticipated, AMPK activity in PLC-SR cells was increased by SCAPi (Fig. 4G). Based on these observations, we reasoned that the activation of AMPK signalling might be involved in promoting autophagy and compromising sorafenib resistance in SCAP knockdown PLC-SR cells. To further confirm this phenomenon, we next treated PLC-SR cells in various conditions with Compound C (CC, 5 μM), an inhibitor of AMPK, and evaluated the levels of AMPK, P62 and LC3. As shown in Fig. S6A-D, CC inhibited autophagy only in the SCAP interference groups (Fig. 4H&I). Furthermore, the data showed that CC had no significant effect on the survival rate of cells with or without sorafenib treatment and SCAP interference, however caused a remarkable increasing survival rate in SCAP-depleted sorafenib-treated HCC cells (Fig. S6E). Colony formation, wound healing and Transwell assays also supported the conclusions (Fig. S6F-I). All these results suggest that sorafenib resistance in HCC induced by SCAP disorder relies on the functional status of AMPK-autophagy signalling.

### SCAP degradation induced by lycorine improves sorafenib resistance in HCC in vitro* and *in vivo

**Fig. 5:**
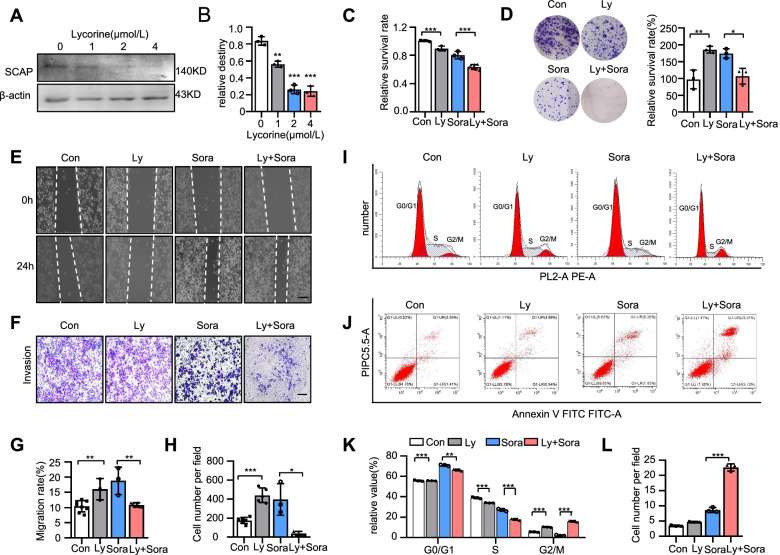
SCAP degradation induced by lycorine improves sorafenib resistance in HCC in vitro. PLC-SR cells (control) were treated with the SCAP inhibitor lycorine at a certain concentration gradient. **A**, **B** Immunoblot analysis of SCAP protein expression at different concentrations (*n* = 3). PLC-SR cells (control) were treated with lycorine (Ly) alone, sorafenib (Sora) alone or a combination of lycorine and sorafenib (Ly + Sora). **C** The viability of sorafenib-treated cells in each group. **D** Clone assays of 4 groups (*n* = 3). Scratch-wound cell migration (**E)**, (**G)** and invasion (**F)**, (**H)** assays for each group (*n* = 3). Flow cytometry analysis of the cell cycle (**I)**, (**K)** and apoptosis (**J**), (**L**) of each group (*n* = 3). Bar = 100 μm. Data are the mean ± SD. *P < 0.05, **P < 0.01, ***P < 0.001. P values were determined by one-way ANOVA in (**B**), (**C**), (**D**), (**G**), (**H**) and (**L**) and repeated-measures ANOVA in (**K**)

As a potent and orally active SCAP inhibitor from the Amaryllidaceae plant, lycorine downregulates SCAP protein levels but does not change its transcription [35]. Lycorine has been experimentally proven to alleviate fat accumulation and metabolic syndrome. Here, we evaluated for the first time the efficacy of SCAP on sorafenib resistance in HCC in vitro and in vivo. Lycorine significantly suppressed the expression of SCAP (up to ~ 75%) in a dose-dependent manner (Fig. 5A-B). Cell viability and proliferation were analysed using CCK-8, clone formation, and cell cycle assays.

Although lycorine alone at the concentration used had little effect on tumour killing, a combination with sorafenib significantly inhibited the proliferation of PLC-SR cells (Fig. 5C-D). Moreover, lycorine combined with sorafenib decelerated the migration and invasion of sorafenib-resistant HCC cells, as shown by wound healing and Transwell assays (Fig. 5E-H), compared with sorafenib or lycorine alone. In addition, flow cytometry analysis indicated that when lycorine was combined with sorafenib, the percentage of S phase cells was decreased, and apoptosis was significantly increased (Fig. 5I-L). All of these results indicate that lycorine reversed acquired resistance to sorafenib in sorafenib-resistant cells.

**Fig. 6:**
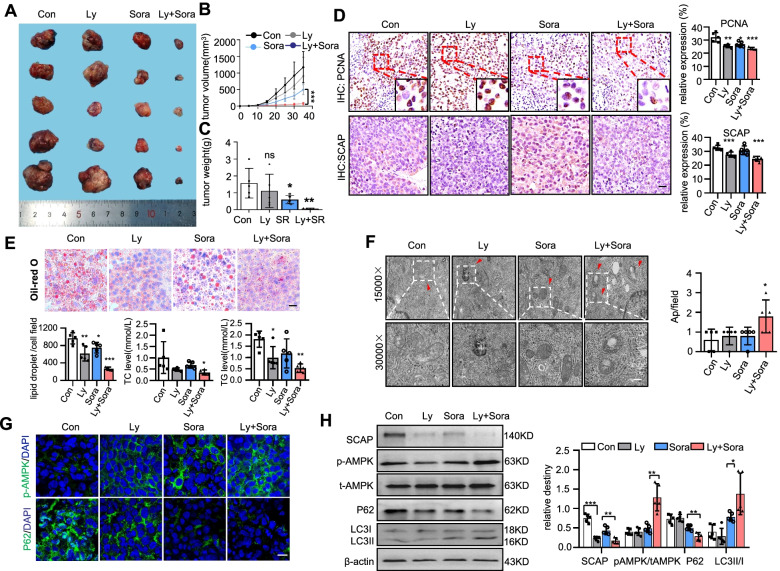
SCAP degradation induced by lycorine improves sorafenib resistance in HCC in vivo. **A** Photographs of subcutaneous tumours after excision (*n* = 5). **B**, **C** Graphs (mean ± SD) showing tumour growth and tumour weight. **D** PCNA and SCAP staining in tumour tissues (*n* = 5). **E** Oil Red O staining in tumour tissues and liver tissues. Bar = 50 μm. The levels of TC and TGs in tumour tissues (*n* = 5). **F** Ultrastructural analysis of tumour tissues from each group. The red arrowhead represents autophagic vacuoles (*n* = 5). Bar = 50 μm. **G** Representative images of immunofluorescence staining of p-AMPK and P62 proteins in each group (*n* = 5). Bar = 100 μm. **H** Immunoblot analysis of SCAP, p-AMPK, t-AMPK, LC3 and P62 protein expression in each group (*n* = 5). Data are the mean ± SD. *P < 0.05, **P < 0.01, ***P < 0.001. P values were determined by one-way ANOVA in (**C**), (**D**), (**E**) and (**F**) and repeated-measures ANOVA in (**B**) and (**H**)

To further support our in vitro findings and to investigate potential clinical applications, we employed an in vivo HCC tumour sphere-bearing mouse model (Fig. S7A). Although the body weight of the mice showed no significant difference in all groups, we observed that the volume of the Ly + Sora and Sora group xenograft tumours was significantly smaller than that of the Con and Ly groups. Moreover, the decrease in the volume of tumours in the Ly + Sora group was more pronounced than that in the Sora alone group (Fig. 6A-C, S7B-D). IHC results showed that SCAP-positive tumour cells tended to have higher PCNA expression in the nucleus (Fig. 6D) and showed the lowest proliferation in the Ly + Sora and Sora groups. Interestingly, lycorine exerted potent antimetastatic effects (Fig. S7E). HE and PCNA IHC staining showed metastatic liver nodules in all groups except for the group with Ly + Sora (Fig. S7F). These data mirror the in vitro effectiveness of lycorine treatments in vivo.

### SCAP degradation induced by lycorine regulates autophagy via AMPK signalling in vivo

We next verified whether the effects observed in the in vitro model were reproduced in vivo. Staining with Oil Red O showed intracellular accumulation of neutral lipids in tumours (Fig. 6E). A higher level of lipid packing was present in the Sora group compared with the Ly + Sora group. This finding may be consistent with the lower levels of SCAP in the Ly + Sora group. The TC and TG levels in tumour cells were in agreement with the ORO prediction described above. We further examined the autophagy level in all groups. TEM showed numerous double-membrane vacuolar structures, which were identified as autophagosomes, existing in the Ly + Sora group but these structures were rarely observed in the other groups (Fig. 6F). Finally, we measured the protein expression of SCAP, AMPK, p-AMPK, P62, and cleaved LC3-II in the tumour tissues by using Western blots and IF staining. The results indicated that treatment with lycorine could promote autophagy through AMPK signalling activation by inhibiting the expression of SCAP (Fig. 6G-H).

**Fig. 7:**
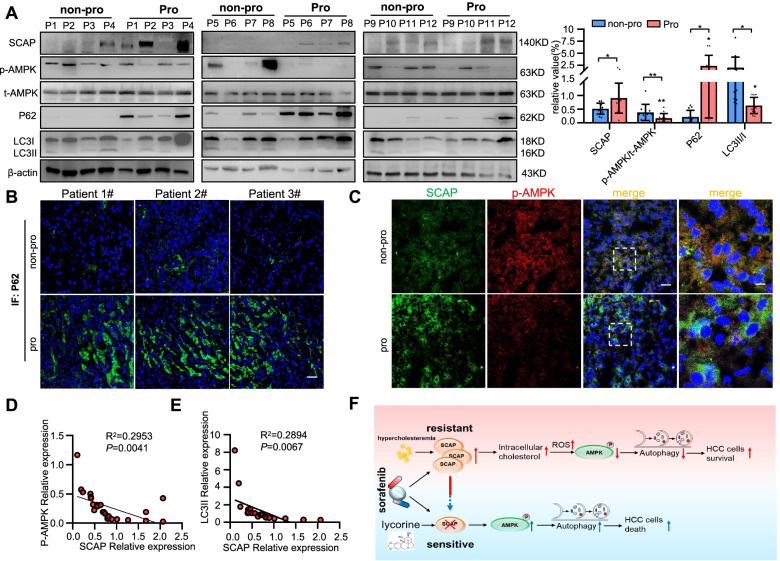
SCAP upregulation promotes sorafenib resistance through inhibiting AMPK-mediated autophagy signalling in HCC tissues. **A** Immunoblot analysis of SCAP, p-AMPK, t-AMPK, LC3 and P62 protein expression in HCC tissues (*n* = 12). **B**, **C** Representative images of immunofluorescence staining of p-AMPK/SCAP and P62 proteins in HCC tissues (*n* = 6). Bar = 100 μm. **D** Correlation analysis of SCAP protein expression and p-AMPK protein expression (*n* = 12). **E** Correlation analysis of SCAP protein expression and LC3-II protein expression (*n* = 12). **F** Model for SCAP-regulated sorafenib resistance in HCC. HCC patients with hyperlipidaemia exhibit resistance to sorafenib, possibly because lipid disorders lead to upregulation of SCAP expression, resulting in stimulation of intracellular cholesterol, high synthesis, and increased uptake, which will result in sorafenib resistance in liver cancer through AMPK-mediated autophagy. Silencing SCAP substantially increases sorafenib-induced cell death. Data are the mean ± SD. *P < 0.05, **P < 0.01, ***P < 0.001. P values were determined by one-way ANOVA in (**A**) and Pearson’s correlation in (**D**) and (**E**)

To test whether the latter finding could be of clinical value, we examined the expression of SCAP, p-AMPK, P62 and LC3-II by IF and Western blots in human HCC tissues. The protein expression of SCAP and P62 was significantly higher and p-AMPK and LC3-II levels were decreased in progressed patients, similar to the in vitro experimental results (Fig. 7A-C). In addition, a negative correlation between SCAP and p-AMPK expression (R^2^ = 0.2953, *P* = 0.004) was observed in 12 paired HCC tissues; a similar correlation was also found between SCAP and LC3-II expression (R^2^ = 0.2894, *P* = 0.007, Fig. 7D-E).

## Discussion

Recent studies have investigated to the linkage between lipid metabolism and sorafenib resistance. Inhibiting fatty acid synthase (FASN) restored the antitumour effect of sorafenib by blocking lipid synthesis [36]. The expression of stearoyl-CoA desaturase-1 (SCD1) had a clinical benefit for sorafenib in HCC patients. When exogenous oleic acid, one of the enzymatic products of SCD1, was added to HCC cells, the effect of an SCD inhibitor on sorafenib sensitization was rescued [37]. However, our retrospective clinical study showed that hypercholesterolaemia but not hypertriglyceridemia contributes to sorafenib resistance, and hypercholesterolaemia may be a candidate biomarker in future HCC patients to predict the likelihood of a response to sorafenib. Because HCC patients with underlying NASH did not benefit from checkpoint inhibition therapy, an interesting strategy is the stepwise accepted stratification of patients who responded well to therapy according to the aetiology of their liver damage and ensuing HCC [38]. Our clinical findings support this notion and provide a theoretical basis for designing personalized treatment options for HCC. However, these data need prospective validation, given the relatively small number of patients in both cohorts. Our following experiment still provides a rationale for this notion.

Liver cancer develops primarily based on chronic inflammation [39]. The latter causes HepG2 cell foam cell formation by disrupting LDLr negative feedback regulation induced by intracellular cholesterol through promotion of SCAP accumulation in the Golgi [34]. Similar results were observed in the PLC cells. Importantly, this phenomenon led to PLC cells being insensitive to sorafenib. Interestingly, SCAP protein expression was significantly higher in HCC tumours and sorafenib-acquired resistant HCC cells. These results indicate that SCAP may be associated with drug resistance to sorafenib. This idea was subsequently confirmed by the following experimental data: SCAP expression was correlated positively with sorafenib sensitivity in five HCC cell lines, sterol-resistant SCAP overexpression reduced HCC cell sensitivity to sorafenib, and reduction of the expression (by siRNA or lycorine) and the biological activity (by DES) of SCAP protein restored PLC-SR cell sensitivity to sorafenib. All this evidence indicates that the biological activity (Golgi accumulation and downstream protein expression) of SCAP is critical for the occurrence of sorafenib resistance.

Drug resistance is a critical issue affecting the outcome of chemotherapy in HCC and is caused either by primary resistance or acquired resistance [40]. Both types consist of a complex mechanism of chemoresistance, including enhanced drug efflux, reduced drug intake, intracellular drug metabolism, activation/inactivation of signalling pathways, alteration of molecular targets, disorder of apoptosis and survival of the cancer cells, and changes in DNA repair machinery [41]. Based on the current state of knowledge about the functional role of SCAP [42, 43], we believe that SCAP affects sorafenib resistance by altering the signalling pathway. Our previous work demonstrated that SCAP negatively regulates AMPK activity by regulating intracellular ROS levels. AMPK activity reduction was already shown to be involved in sorafenib resistance [44]. In sorafenib-resistant HCC cells, activation of the AMPK pathway achieved the sensitization of HCC to sorafenib treatment [45]. Here, we found that this pathway in sorafenib-resistant cells or in vivo was defective and was rescued by SCAP knockdown, suggesting that increased SCAP inhibited the AMPK pathway. Rescue experiments using Compound C, an inhibitor of AMPK, in SCAP-depleted sorafenib-resistant HCC cells restored their resistance to sorafenib. Because it is reported that Compound C affected autophagy by mTOR-dependent [46] or independent pathway [47], and mTOR may influence cell growth [48]. We used chloroquine in SCAP-depleted sorafenib-treated HCC cells and Compound C to treat different groups of PLC-SR cells. Results showed that Compound C reduced sensitivity of sorafenib only if autophagy is inhibited. Thus, in our view, this regulatory role of SCAP in AMPK-autophagy signalling is a potential mechanism for sorafenib resistance.

The AMPK pathway is an important upstream signal of autophagic activation. Activated AMPK/mTOR signalling promotes the sensitization of HCC to sorafenib via autophagic regulation [49]. The effects of sorafenib resistance disappeared or diminished, accompanied by restoration of autophagic activity, during the suppression of SCAP via either pharmacological inhibitors (lycorine) or gene silencing. Collectively, enhanced SCAP signalling leads to decreased AMPK activity and increased autophagy, which might be a novel mechanism of acquired resistance to sorafenib. Although this phenomenon provides a tantalizing explanation for our findings, it may not be the only mechanism underlying these changes. On the basis of our previous study, we also believe that SCAP may be involved in the regulation of sorafenib resistance through other mechanisms, such as through the regulation of tumour angiogenesis (VEGFR) [50]. Regardless, we present new insights into and abundant evidence of the clinical characteristics of sorafenib-resistant HCC patients. Hypercholesterolaemia should be considered together with the current list of risks and benefits of sorafenib medication to guide clinical decisions.

Because SCAP plays a pivotal role in the regulation of cholesterol homeostasis [51], targeting it continuously could be an attractive strategy for the treatment of metabolic diseases [52–54]. To date, a series of SCAP inhibitors, including cholesterol, fatostatin, and botulin, have been reported [52, 55, 56]. The symptoms of metabolic diseases, such as obesity, hyperlipidaemia, and insulin resistance, are alleviated by these compounds, yet the accompanying ER stress constrains their wide usage [57–59]. In contrast to most other SCAP inhibitors, lycorine downregulates SCAP expression but does not induce ER stress. Lycorine has been shown to prevent the growth and metastasis of hormone-refractory prostate cancer and melanoma C8161 cell-dominant vasculogenic mimicry [60]. This molecule was also shown to increase liver cancer cell sensitivity to sorafenib in the present study. Notably, lycorine promoted the degradation of SCAP by transferring it to lysosomes in an autophagy-independent pathway. Hence, the changes in autophagic flux that we observed were caused by the absence of the SCAP protein rather than the direct effects of lycorine. This phenomenon will reduce potential confusion without affecting the interpretation of study findings, increasing the objectivity, reliability, and reproducibility of the research conclusions. The present results provide evidence supporting lycorine as a potential anticancer drug that can enhance the efficacy of sorafenib to treat HCC, particularly sorafenib-resistant HCC, which contributes to further clinical investigation.

## Conclusions

In summary, we uncovered an important role of SCAP in regulating HCC sorafenib resistance (Fig. 7F). Our study demonstrated that SCAP contributes to sorafenib resistance through AMPK-mediated autophagic regulation. More importantly, we applied a combination of a SCAP inhibitor with sorafenib for HCC treatment and observed significant sensitization of the cells to sorafenib. Our study presents new insights into and abundant evidence of the clinical characteristics of sorafenib-resistant HCC patients and provides a novel therapeutic strategy to enhance the treatment response in sorafenib-resistant HCC.

## Supplementary Information

**Additional file 1:** **Fig. S1.** SCAP protein expression is related to the sensitivity to sorafenib in different HCC cells. **Fig. S2.** Sterol-resistant SCAP overexpression triggers sensitivity to sorafenib in an HCC cell line. **Fig. S3.** Inhibition of Golgi *translocation* of SCAP reverses sorafenib resistance in HCC cell lines. **Fig. S4.** Sorafenib kills cells by autophagic activation. **Fig. S5.** The mRNA levels of autophagy-related genes were detected by qRT-PCR. **Fig. S6.** Compound C promotes cell proliferation in SCAP-depleted sorafenib-treated HCC cells. **Fig. S7.** Establishment of the HCC tumour-bearing mouse models.

## Data Availability

The extra-material is available in the supplemental material of the journal.

## Abbreviations

ABCC2: ATP-binding cassette transporter C2
AMPK: AMP-activated protein kinase
BMI: Body Mass Index
CC: Compound C
DES: Desmosterol
ER: Endoplasmic reticulum
FASN: Fatty acid synthase
HCC: Hepatocellular carcinoma
HDL: High-density lipoprotein
HLP: Hyperlipidemia
HMGCR: Hydroxy-3-methylglutaryl-coenzyme A reductase
IL-1β: Interleukin-1β
LC3: Microtubule-associated protein 1 light chain 3
LDL: Low Density Lipoprotein
mTOR: Rapamycin
NASH: Non-alcoholic steatohepatitis
OCT1: Octamer transcription factor 1
OS: Overall survival
P62: Sequestosome 1
PCNA: Proliferation Cell Nuclear Antigen
ROS: Reactive oxygen species
SCAP: Sterol-regulatory element binding proteins cleavage-activating protein
SCD1: Stearoyl-CoA desaturase-1
SR: Sorafenib resistant
SREBP: Sterol-regulatory element binding proteins
TC: Total cholesterol
TEM: Transmission electron microscopy
TG: Triglyceride
TKI: Tyrosine kinase inhibitor
VEGFR: Vascular endothelial growth factor receptors

## References

[1] Wilhelm SM, Carter C, Tang L, Wilkie D, McNabola A, Rong H, Chen C (2004). BAY 43–9006 exhibits broad spectrum oral antitumor activity and targets the RAF/MEK/ERK pathway and receptor tyrosine kinases involved in tumor progression and angiogenesis. Cancer Res.

[2] Llovet JM, Ricci S, Mazzaferro V, Hilgard P, Gane E, Blanc JF, de Oliveira AC (2008). Sorafenib in advanced hepatocellular carcinoma. N Engl J Med.

[3] Cheng AL, Kang YK, Chen Z, Tsao CJ, Qin S, Kim JS, Luo R (2009). Efficacy and safety of sorafenib in patients in the Asia-Pacific region with advanced hepatocellular carcinoma: a phase III randomised, double-blind, placebo-controlled trial. Lancet Oncol.

[4] Chen J, Jin R, Zhao J, Liu J, Ying H, Yan H, Zhou S (2015). Potential molecular, cellular and microenvironmental mechanism of sorafenib resistance in hepatocellular carcinoma. Cancer Lett.

[5] Villanueva A, Llovet JM (2012). Second-line therapies in hepatocellular carcinoma: emergence of resistance to sorafenib. Clin Cancer Res.

[6] Weingärtner O, Böhm M, Laufs U (2009). Controversial role of plant sterol esters in the management of hypercholesterolaemia. Eur Heart J.

[7] Nelson ER, Wardell SE, Jasper JS, Park S, Suchindran S, Howe MK, Carver NJ (2013). 27-Hydroxycholesterol links hypercholesterolemia and breast cancer pathophysiology. Science.

[8] Plotti F, Terranova C, Luvero D, Bartolone M, Messina G, Feole L, Cianci S (2020). Diet and chemotherapy: the effects of fasting and ketogenic diet on cancer treatment. Chemotherapy.

[9] Pelosof LC, Gerber DE (2010). Paraneoplastic syndromes: an approach to diagnosis and treatment. Mayo Clin Proc.

[10] Zhang X, Coker OO, Chu ES, Fu K, Lau HCH, Wang YX, Chan AWH (2021). Dietary cholesterol drives fatty liver-associated liver cancer by modulating gut microbiota and metabolites. Gut.

[11] Kim GA, Shim JJ, Lee JS, Kim BH, Kim JW, Oh CH, Oh CM (2019). Effect of statin use on liver cancer mortality considering hypercholesterolemia and obesity in patients with non-cirrhotic chronic hepatitis B. Yonsei Med J.

[12] Mohammad N, Malvi P, Meena AS, Singh SV, Chaube B, Vannuruswamy G, Kulkarni MJ (2014). Cholesterol depletion by methyl-β-cyclodextrin augments tamoxifen induced cell death by enhancing its uptake in melanoma. Mol Cancer.

[13] Mohammad N, Singh SV, Malvi P, Chaube B, Athavale D, Vanuopadath M, Nair SS (2015). Strategy to enhance efficacy of doxorubicin in solid tumor cells by methyl-β-cyclodextrin: Involvement of p53 and Fas receptor ligand complex. Sci Rep.

[14] Benegiamo G, Mure LS, Erikson G, Le HD, Moriggi E, Brown SA, Panda S (2018). The RNA-binding protein NONO coordinates hepatic adaptation to feeding. Cell Metab.

[15] Sinha RA, Bruinstroop E, Singh BK, Yen PM (2019). Nonalcoholic fatty liver disease and hypercholesterolemia: roles of thyroid hormones, metabolites, and agonists. Thyroid.

[16] Lee SH, Lee JH, Im SS (2020). The cellular function of SCAP in metabolic signaling. Exp Mol Med.

[17] Sakai J, Rawson RB (2001). The sterol regulatory element-binding protein pathway: control of lipid homeostasis through regulated intracellular transport. Curr Opin Lipidol.

[18] Durst R, Jansen A, Erez G, Bravdo R, Butbul E, Ben Avi L, Shpitzen S (2006). The discrete and combined effect of SREBP-2 and SCAP isoforms in the control of plasma lipids among familial hypercholesterolaemia patients. Atherosclerosis.

[19] Lim SA, Wei J, Nguyen TM, Shi H, Su W, Palacios G, Dhungana Y (2021). Lipid signalling enforces functional specialization of T(reg) cells in tumours. Nature.

[20] Schaeffeler E, Hellerbrand C, Nies AT, Winter S, Kruck S, Hofmann U, van der Kuip H (2011). DNA methylation is associated with downregulation of the organic cation transporter OCT1 (SLC22A1) in human hepatocellular carcinoma. Genome Med.

[21] Wen X, Joy MS, Aleksunes LM (2017). In vitro transport activity and trafficking of MRP2/ABCC2 polymorphic variants. Pharm Res.

[22] Shen M, Lin L (2019). Functional variants of autophagy-related genes are associated with the development of hepatocellular carcinoma. Life Sci.

[23] Méndez-Blanco C, Fondevila F, García-Palomo A, González-Gallego J, Mauriz JL (2018). Sorafenib resistance in hepatocarcinoma: role of hypoxia-inducible factors. Exp Mol Med.

[24] Beloribi-Djefaflia S, Vasseur S, Guillaumond F (2016). Lipid metabolic reprogramming in cancer cells. Oncogenesis.

[25] Heqing Y, Bin L, Xuemei Y, Linfa L (2016). The role and mechanism of autophagy in sorafenib targeted cancer therapy. Crit Rev Oncol Hematol.

[26] Park MA, Reinehr R, Häussinger D, Voelkel-Johnson C, Ogretmen B, Yacoub A, Grant S (2010). Sorafenib activates CD95 and promotes autophagy and cell death via Src family kinases in gastrointestinal tumor cells. Mol Cancer Ther.

[27] Lin CI, Whang EE, Lorch JH, Ruan DT (2012). Autophagic activation potentiates the antiproliferative effects of tyrosine kinase inhibitors in medullary thyroid cancer. Surgery.

[28] Singh R, Kaushik S, Wang Y, Xiang Y, Novak I, Komatsu M, Tanaka K (2009). Autophagy regulates lipid metabolism. Nature.

[29] Singh R, Cuervo AM (2011). Autophagy in the cellular energetic balance. Cell Metab.

[30] Hu X, Lu Z, Yu S, Reilly J, Liu F, Jia D, Qin Y (2019). CERKL regulates autophagy via the NAD-dependent deacetylase SIRT1. Autophagy.

[31] Li D, Chen A, Lan T, Zou Y, Zhao L, Yang P, Qu H (2019). SCAP knockdown in vascular smooth muscle cells alleviates atherosclerosis plaque formation via up-regulating autophagy in ApoE(-/-) mice. Faseb j.

[32] Dasgupta S, Putluri N, Long W, Zhang B, Wang J, Kaushik AK, Arnold JM (2015). Coactivator SRC-2-dependent metabolic reprogramming mediates prostate cancer survival and metastasis. J Clin Invest.

[33] Korn BS, Shimomura I, Bashmakov Y, Hammer RE, Horton JD, Goldstein JL, Brown MS (1998). Blunted feedback suppression of SREBP processing by dietary cholesterol in transgenic mice expressing sterol-resistant SCAP(D443N). J Clin Invest.

[34] Chen Y, Ruan XZ, Li Q, Huang A, Moorhead JF, Powis SH, Varghese Z (2007). Inflammatory cytokines disrupt LDL-receptor feedback regulation and cause statin resistance: a comparative study in human hepatic cells and mesangial cells. Am J Physiol Renal Physiol.

[35] Zheng ZG, Zhu ST, Cheng HM, Zhang X, Cheng G, Thu PM, Wang SP (2021). Discovery of a potent SCAP degrader that ameliorates HFD-induced obesity, hyperlipidemia and insulin resistance via an autophagy-independent lysosomal pathway. Autophagy.

[36] Sounni NE, Cimino J, Blacher S, Primac I, Truong A, Mazzucchelli G, Paye A (2014). Blocking lipid synthesis overcomes tumor regrowth and metastasis after antiangiogenic therapy withdrawal. Cell Metab.

[37] Ma MKF, Lau EYT, Leung DHW, Lo J, Ho NPY, Cheng LKW, Ma S (2017). Stearoyl-CoA desaturase regulates sorafenib resistance via modulation of ER stress-induced differentiation. J Hepatol.

[38] Pfister D, Núñez NG, Pinyol R, Govaere O, Pinter M, Szydlowska M, Gupta R (2021). NASH limits anti-tumour surveillance in immunotherapy-treated HCC. Nature.

[39] Nguyen PHD, Ma S, Phua CZJ, Kaya NA, Lai HLH, Lim CJ, Lim JQ (2021). Intratumoural immune heterogeneity as a hallmark of tumour evolution and progression in hepatocellular carcinoma. Nat Commun.

[40] Marin JJ, Romero MR, Briz O (2010). Molecular bases of liver cancer refractoriness to pharmacological treatment. Curr Med Chem.

[41] Cabral LKD, Tiribelli C, Sukowati CHC (2020). Sorafenib resistance in hepatocellular carcinoma: the relevance of genetic heterogeneity. Cancers (Basel).

[42] Li D, Liu M, Li Z, Zheng G, Chen A, Zhao L, Yang P (2021). Sterol-resistant SCAP overexpression in vascular smooth muscle cells accelerates atherosclerosis by increasing local vascular inflammation through activation of the NLRP3 inflammasome in mice. Aging Dis.

[43] Li LC, Varghese Z, Moorhead JF, Lee CT, Chen JB, Ruan XZ (2013). Cross-talk between TLR4-MyD88-NF-κB and SCAP-SREBP2 pathways mediates macrophage foam cell formation. Am J Physiol Heart Circ Physiol.

[44] Wang K, Zhang Z, Tsai HI, Liu Y, Gao J, Wang M, Song L (2021). Branched-chain amino acid aminotransferase 2 regulates ferroptotic cell death in cancer cells. Cell Death Differ.

[45] Zhu Y, Xu J, Hu W, Wang F, Zhou Y, Xu W, Gong W (2020). TFAM depletion overcomes hepatocellular carcinoma resistance to doxorubicin and sorafenib through AMPK activation and mitochondrial dysfunction. Gene.

[46] Vucicevic L, Misirkic M, Janjetovic K, Vilimanovich U, Sudar E, Isenovic E, Prica M (2011). Compound C induces protective autophagy in cancer cells through AMPK inhibition-independent blockade of Akt/mTOR pathway. Autophagy.

[47] Zhao X, Luo G, Cheng Y, Yu W, Chen R, Xiao B, Xiang Y (2018). Compound C induces protective autophagy in human cholangiocarcinoma cells via Akt/mTOR-independent pathway. J Cell Biochem.

[48] Du L, Li X, Zhen L, Chen W, Mu L, Zhang Y, Song A (2018). Everolimus inhibits breast cancer cell growth through PI3K/AKT/mTOR signaling pathway. Mol Med Rep.

[49] Li J, Wu PW, Zhou Y, Dai B, Zhang PF, Zhang YH, Liu Y (2018). Rage induces hepatocellular carcinoma proliferation and sorafenib resistance by modulating autophagy. Cell Death Dis.

[50] Li Z, Li D, Rao Y, Wei L, Liu M, Zheng G, Yao Y (2021). SCAP knockout in SM22α-Cre mice induces defective angiogenesis in the placental labyrinth. Biomed Pharmacother.

[51] Stewart EV, Nwosu CC, Tong Z, Roguev A, Cummins TD, Kim DU, Hayles J (2011). Yeast SREBP cleavage activation requires the Golgi Dsc E3 ligase complex. Mol Cell.

[52] Tang JJ, Li JG, Qi W, Qiu WW, Li PS, Li BL, Song BL (2011). Inhibition of SREBP by a small molecule, betulin, improves hyperlipidemia and insulin resistance and reduces atherosclerotic plaques. Cell Metab.

[53] Moon YA, Liang G, Xie X, Frank-Kamenetsky M, Fitzgerald K, Koteliansky V, Brown MS (2012). The Scap/SREBP pathway is essential for developing diabetic fatty liver and carbohydrate-induced hypertriglyceridemia in animals. Cell Metab.

[54] Loregger A, Raaben M, Nieuwenhuis J, Tan JME, Jae LT, van den Hengel LG, Hendrix S (2020). Haploid genetic screens identify SPRING/C12ORF49 as a determinant of SREBP signaling and cholesterol metabolism. Nat Commun.

[55] Kamisuki S, Mao Q, Abu-Elheiga L, Gu Z, Kugimiya A, Kwon Y, Shinohara T (2009). A small molecule that blocks fat synthesis by inhibiting the activation of SREBP. Chem Biol.

[56] Radhakrishnan A, Ikeda Y, Kwon HJ, Brown MS, Goldstein JL (2007). Sterol-regulated transport of SREBPs from endoplasmic reticulum to Golgi: oxysterols block transport by binding to Insig. Proc Natl Acad Sci U S A.

[57] Devries-Seimon T, Li Y, Yao PM, Stone E, Wang Y, Davis RJ, Flavell R (2005). Cholesterol-induced macrophage apoptosis requires ER stress pathways and engagement of the type A scavenger receptor. J Cell Biol.

[58] Hager L, Li L, Pun H, Liu L, Hossain MA, Maguire GF, Naples M (2012). Lecithin:cholesterol acyltransferase deficiency protects against cholesterol-induced hepatic endoplasmic reticulum stress in mice. J Biol Chem.

[59] Sozen E, Ozer NK (2017). Impact of high cholesterol and endoplasmic reticulum stress on metabolic diseases: An updated mini-review. Redox Biol.

[60] Liu R, Cao Z, Tu J, Pan Y, Shang B, Zhang G, Bao M (2012). Lycorine hydrochloride inhibits metastatic melanoma cell-dominant vasculogenic mimicry. Pigment Cell Melanoma Res.

