# Supplementary material for: Cholesterol sensor SCAP contributes to sorafenib resistance by regulating autophagy in hepatocellular carcinoma
Source: J Exp Clin Cancer Res. 2022 Mar 30;41:116. doi: 10.1186/s13046-022-02306-4 (PMC8966370; doi:10.1186/s13046-022-02306-4)
Supplement: Supplementary file 1 — Additional file 1: Fig. S1. SCAP protein expression is related to the sensitivity to sorafenib in different HCC cells. Fig. S2. Sterol-resistant SCAP overexpression triggers sensitivity to sorafenib in an HCC cell line. Fig. S3. Inhibition of Golgi translocation of SCAP reverses sorafenib resistance in HCC cell lines. Fig. S4. Sorafenib kills cells by autophagic activation. Fig. S5. The mRNA levels of autophagy-related genes were detected by qRT-PCR. Fig. S6. Compound C promotes cell proliferation in SCAP-depleted sorafenib-treated HCC cells. Fig. S7. Establishment of the HCC tumour-bearing mouse models. [file 13046_2022_2306_MOESM1_ESM.docx]

**Cholesterol sensor SCAP contributes to Sorafenib resistance by regulating autophagy in hepatocellular carcinoma**

Danyang Li, Yingcheng Yao, Yuhan Rao, Xinyu Huang, Li Wei, Zhimei You, Gou Zheng, Xiaoli Hou, Yu Su, Zac Varghese, John F. Moorhead, Yaxi Chen and Xiong Z Ruan

**Supplementary Figures**

**Supplementary Figure 1**

**
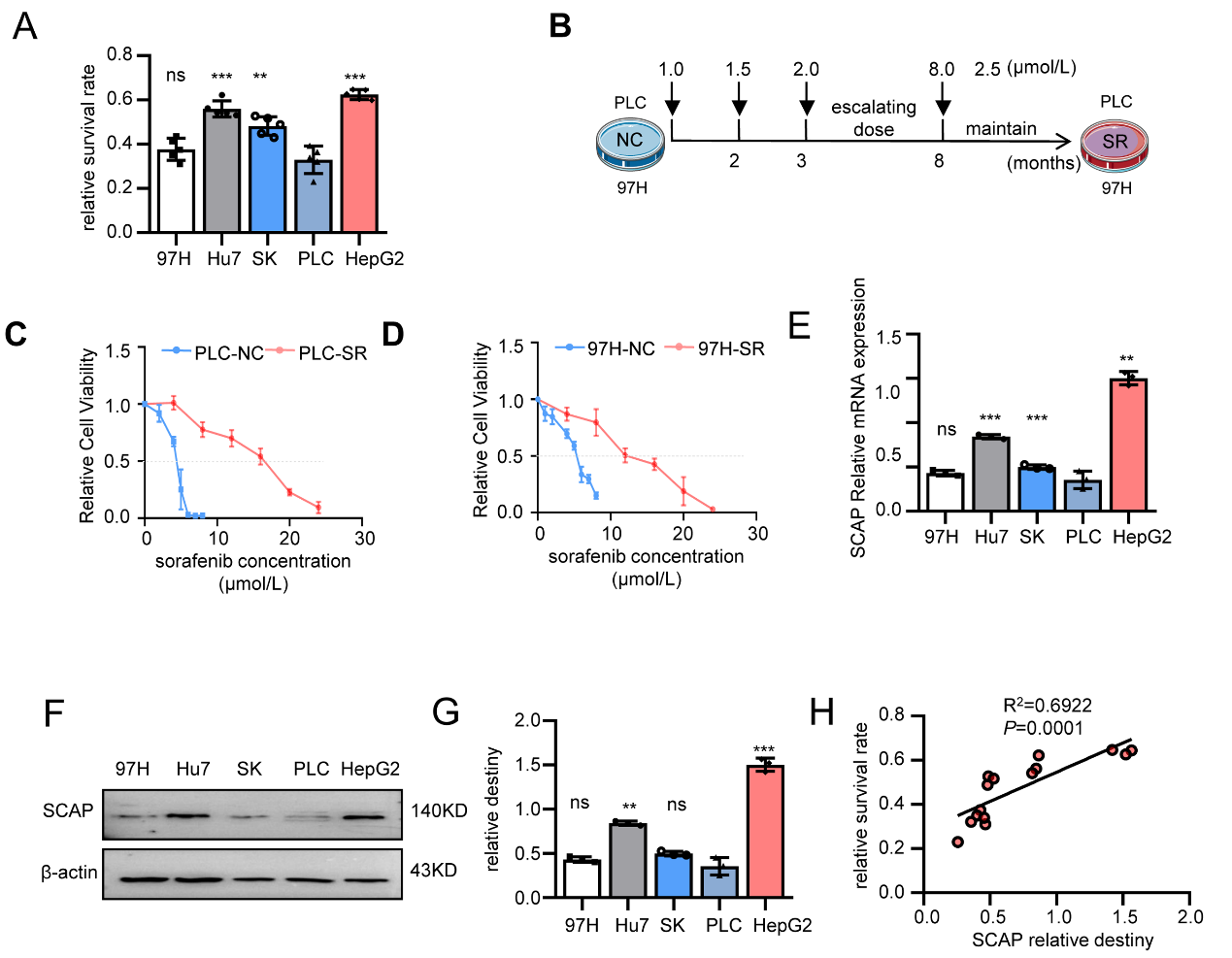
**

**Fig. S1: SCAP protein expression is related to the sensitivity to sorafenib in different HCC cells.** (A) The sensitivity of 5 different HCC cell lines to sorafenib (n=5). (B) Sorafenib-resistant clones were established by subjecting PLC/PRF/5 and MHCC-97H cells to continuous administration of gradually increasing sorafenib concentrations. Identification of the resistance index of sorafenib-resistant PLC/PRF/5 cells (C) and MHCC-97H cells (D) (n=3). (E) mRNA expression of SCAP as measured by qRT-PCR (n=3). (F) Immunoblot analysis of SCAP protein expression (n=3). (G) The histogram represents the relative expression of SCAP. (H) Correlation analysis of SCAP protein expression and the sensitivity of 5 HCC cell lines to sorafenib (n=3). Data are the mean ± SD. *P< 0.05, **P< 0.01, ***P< 0.001. P values were determined by one-way ANOVA in (A), (F), and (G); repeated-measures ANOVA in (C) and (D); and Pearson’s correlation in (H).

**Supplementary Figure 2**


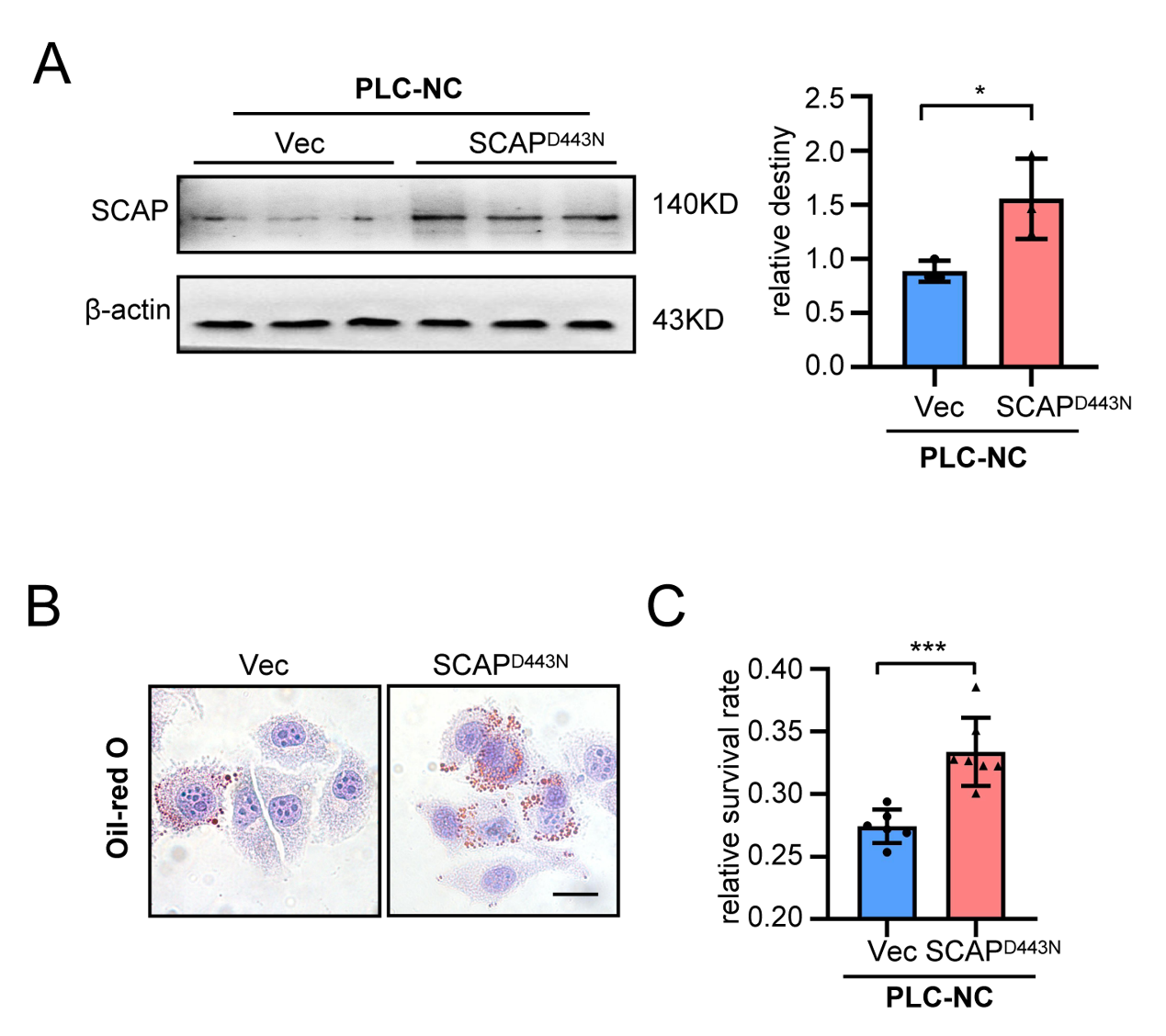


**Fig. S2: Sterol-resistant SCAP overexpression triggers sensitivity to sorafenib in an HCC cell line.** The plasmid p-TK-HSV-SCAP (D443N) was transfected into cells. (A) Immunoblot analysis of SCAP protein expression in vector and SCAP D443N-mutated cells (n=3). (B) Representative images of vector and SCAP D443N-mutated cells stained with Oil Red O (n=4). Bar=50 µm. (C) The viability of sorafenib-treated vector and SCAP D443N-mutated cells. Data are the mean ± SD. *P< 0.05, **P< 0.01, ***P< 0.001. P values were determined by Student’s t test.

**Supplementary Figure 3**


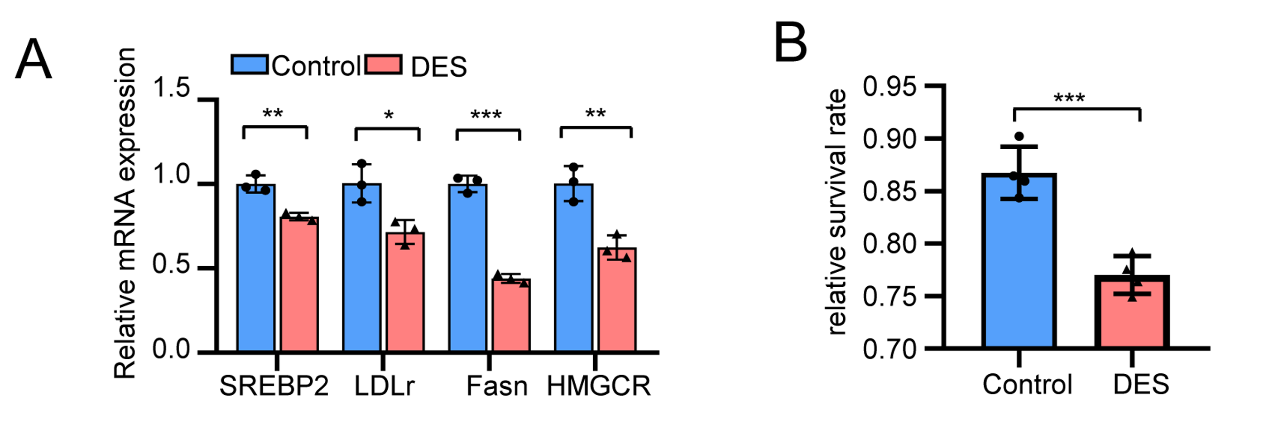


**Fig. S3: Inhibition of Golgi translocation of SCAP reverses sorafenib resistance in HCC cell lines.** PLC-SR cells were pretreated with the SCAP translocation inhibitor desmosterol (DES) (5 µm/L) for 24 h. (A) mRNA expression of SCAP downstream genes (SREBP2, LDLr, Fasn, HMGCR) as measured by qRT-PCR (n=3) in the control group and DES group (n=3). (B) The viability of sorafenib-treated cells in each group (n=3). Data are the mean ± SD. *P< 0.05, **P< 0.01, ***P< 0.001. P values were determined by repeated-measures ANOVA in (A) and Student’s t test in (B).

**Supplementary Figure 4**


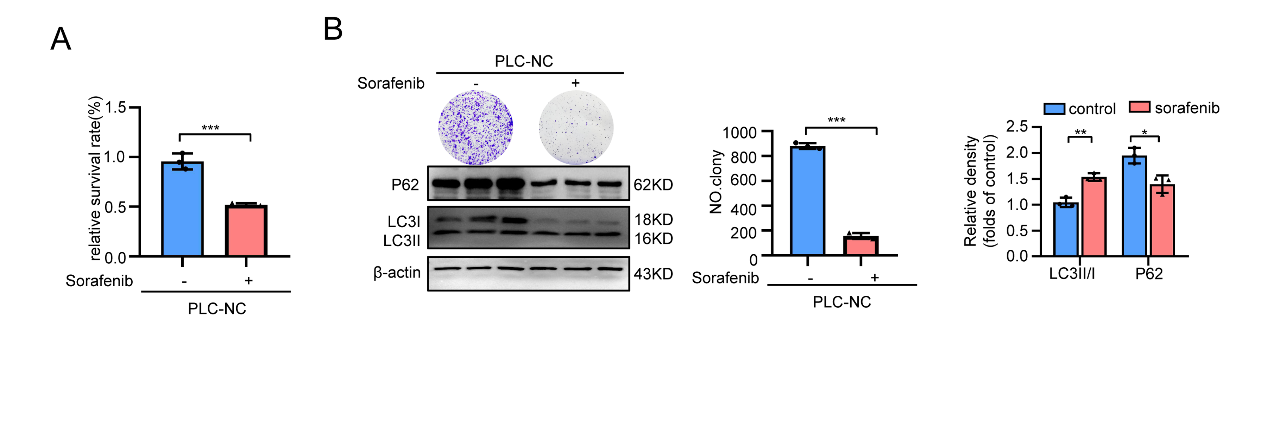


**Fig. S4: Sorafenib kills cells by autophagic activation.** (A) The viability of NC cells with or without sorafenib treatment (n=3). (B) Clone assays of cells and Immunoblot analysis of LC3 and P62 protein expression in NC cells with or without sorafenib treatment (n=3). Data are the mean ± SD. *P< 0.05, **P< 0.01, ***P< 0.001. P values were determined by Student’s t test.

**Supplementary Figure 5**


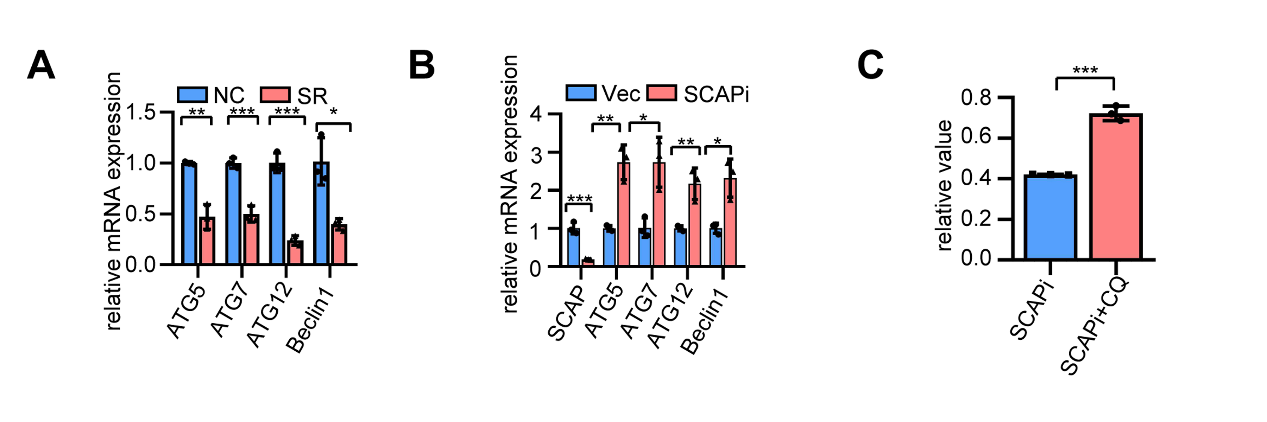


**Fig. S5: The mRNA levels of autophagy-related genes were detected by qRT-PCR.** (A), (B) mRNA expression of autophagy-related genes (ATG5, ATG7, ATG12, Beclin1) as measured by qRT-PCR (n=3) in each group (n=3). SCAP-depleted sorafenib-treated HCC cells were treated with chloroquine (20μM). (C) The viability of the sorafenib-treated SCAPi cells and SCAPi + CQ cells (n=3). Data are the mean ± SD. *P< 0.05, **P< 0.01, ***P< 0.001. P values were determined by repeated-measures ANOVA in (A) and (B) and Student’s t test in (C).

**Supplementary Figure 6**


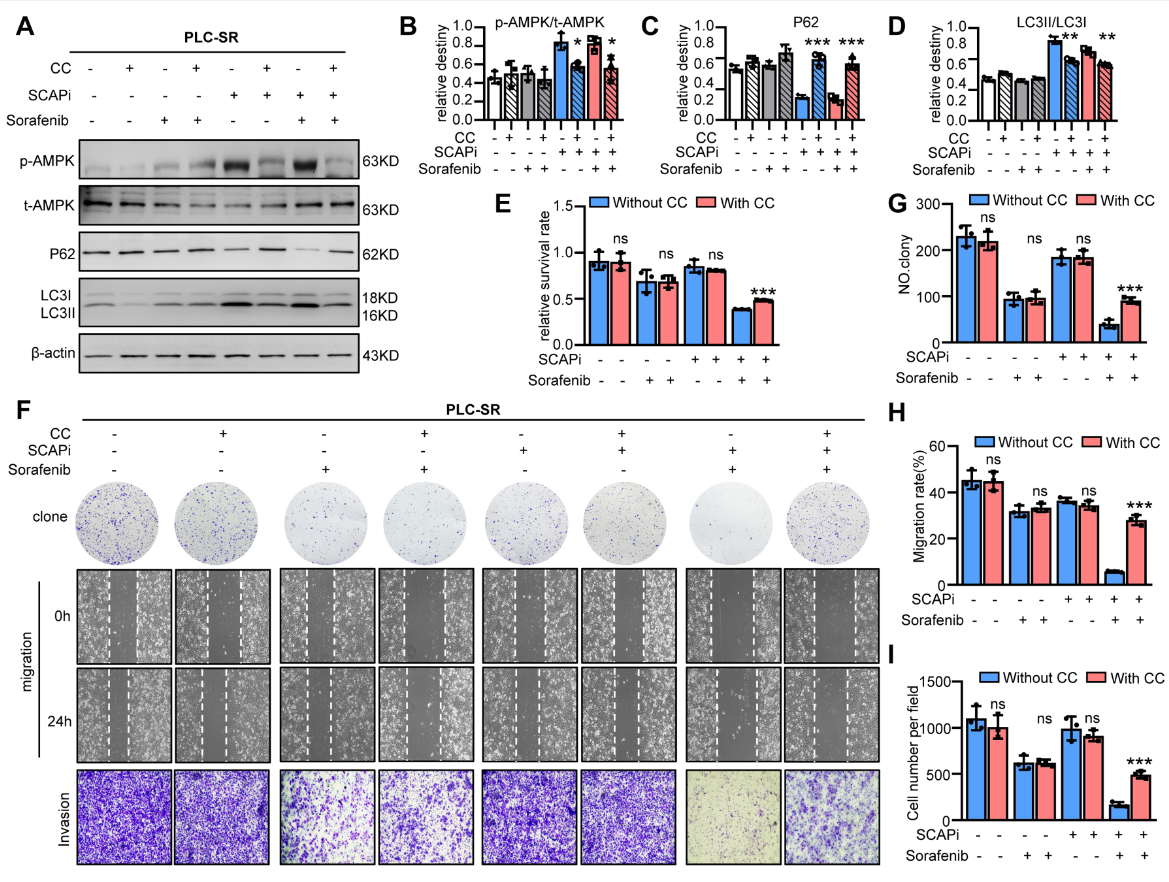


**Fig. S6: Compound C promotes cell proliferation in SCAP-depleted sorafenib-treated HCC cells.** PLC-SR cells in different groups were treated with or without compound C (n=3). (A), (B), (C), (D) Immunoblot analysis of p-AMPK, t-AMPK, LC3 and P62 protein expression in 8 groups (n=3). (E) The viability of 8 group cells (n=3). (F) Clone assays (G), Scratch-wound cell migration assays (H) and invasion assays (I) of 8 groups in PLC-SR cells (n=3). Data are the mean ± SD. *P< 0.05, **P< 0.01, ***P< 0.001. P values were determined by Student’s t test.

**Supplementary Figure 7**


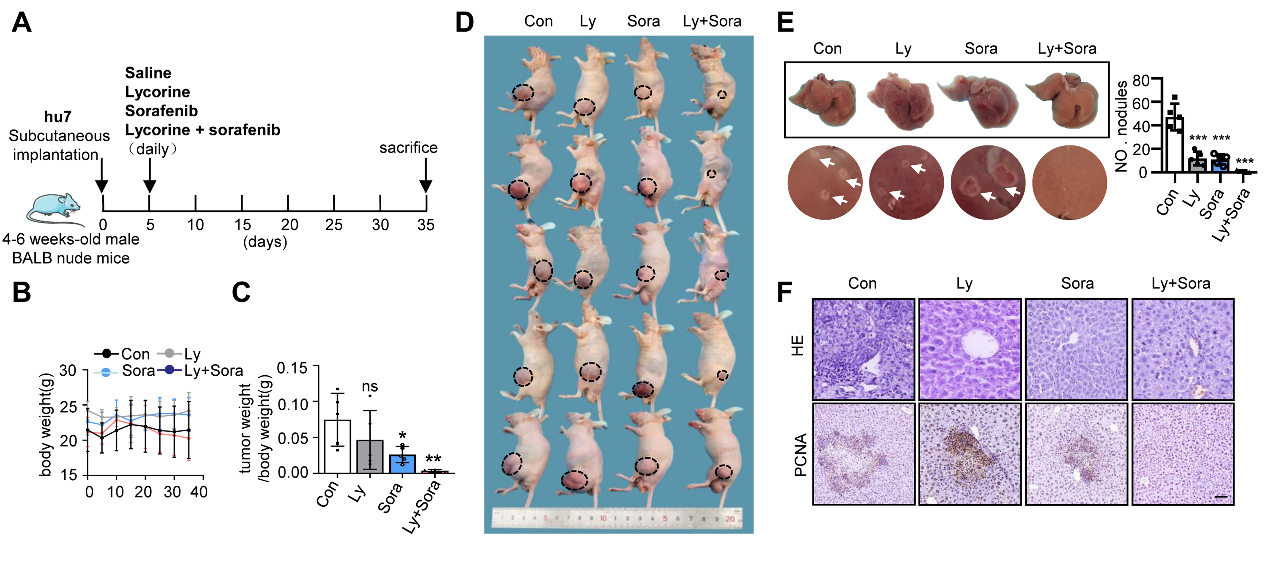


**Fig. S7: Establishment of the HCC tumour-bearing mouse models.** (A) Hu7 cells (2 × 10^6^ per site) were subcutaneously injected into the flanks of nude mice (n =5 per group) and were treated with saline (50 µL/day), lycorine (10 mg/kg/day), sorafenib (30 mg/kg/day) or both. (B), (C) Graphs (mean ± SD) showing body weight and the ratio of tumour weight to body weight (n =5). (D) Representative tumour-bearing mice of the subcutaneous tumour model treated as indicated (n=5). (E) Representative images of the metastasized livers in each group after 35 days (n=5). The white arrowhead represents metastases. (F) HE and PCNA staining in livers. Bar=50 µm. Data are the mean ± SD. *P< 0.05, **P< 0.01, ***P< 0.001. P values were determined by repeated-measures ANOVA in (B) and one-way ANOVA in (C).
